# Assessing access to care in a postpartum pelvic floor healing clinic

**DOI:** 10.3389/fruro.2025.1548341

**Published:** 2025-04-16

**Authors:** Anya Singh-Varma, Li Wang, Rachel Durst, Pamela A. Moalli, Lauren E. Giugale

**Affiliations:** ^1^ School of Medicine, University of Pittsburgh, Pittsburgh, PA, United States; ^2^ Clinical and Translational Science Institute University of Pittsburgh, Pittsburgh, PA, United States; ^3^ Department of Obstetrics, Gynecology and Reproductive Sciences, UPMC Magee-Womens Hospital, Pittsburgh, PA, United States; ^4^ Magee-Womens Research Institute, Pittsburgh, PA, United States

**Keywords:** area deprivation index, access to care, postpartum, health equity, median income, disparities, perineal laceration, obstetric anal sphincter injuries

## Abstract

**Introduction:**

Disparities in pregnancy care exist in the United States, with limited data on access to specialized postpartum care for patients with complicated perineal lacerations. Our objective was to assess for disparities in access to a postpartum pelvic floor healing clinic following vaginal delivery. We hypothesized an underrepresentation of patients from more resource-deprived neighborhoods and those with longer travel times to the specialized clinic.

**Methods:**

This is a retrospective cohort study comparing sociodemographic variables from a historical cohort of patients with third- and fourth-degree lacerations following vaginal delivery to a cohort of patients evaluated in a postpartum pelvic floor healing clinic. The primary outcome involved the comparison of the neighborhood area deprivation index between groups. The secondary outcomes included median household income, driving time, and distance to the hospital.

**Results:**

Patients seen in the postpartum pelvic floor healing clinic were older (31.3 vs. 29.9 years, *p* < 0.01) and more likely to be multiparous (20.3% vs. 13.1%, *p* = 0.04). Race, ethnicity, and operative vaginal delivery were similar between groups. Patients from the postpartum pelvic floor healing clinic had more postpartum visits [3 (IQR 2–4) vs. 2 (IQR 1–2) visits, *p* < 0.01]. There was no significant difference in median neighborhood area deprivation indices [4 (IQR 2–7) vs. 5 (IQR 3–7), *p* = 0.06]. Fewer patients from the most resource-deprived neighborhoods were seen in the postpartum pelvic floor healing clinic, though this was not statistically significant (4.5% vs. 8.9%, *p* = 0.06). There were no significant differences in median household income or driving distance to the hospital between groups.

**Conclusions:**

Access to a specialized postpartum pelvic floor healing clinic at our institution appears equitable across several sociodemographic factors.

## 1 Introduction

Vaginal delivery is the most common cause of perineal trauma, and approximately 70%–80% of patients undergoing vaginal childbirth will experience a laceration or tear to the vagina or perineum. Furthermore, 2%–6% of patients experience more complex third- or fourth-degree tears, also known as obstetric anal sphincter injuries (OASIs) (1, 2). OASIs are associated with an increased risk of complications including wound breakdown, infection, anal sphincter incontinence, pain, and pelvic floor dysfunction (3). Additionally, OASIs pose significant psychological stress to patients (4). Thus, there has been heightened emphasis on the importance of early postpartum follow-up and close monitoring of individuals who have experienced OASIs (5–7). As such, a specialized postpartum pelvic floor healing clinic (PPFHC) was established at our institution to provide the necessary and complex care required for patients with OASIs.

In the United States, there are ongoing disparities in maternal healthcare ranging from inadequate access to maternal mortality (8–10). There has been an increase in the number of publications assessing how neighborhood-level disparities relate to access to healthcare and healthcare disparities (11–13), and there are now multiple tools to help quantify and understand this relationship (14–19). With our current knowledge of existing and pervasive inequities in the care of pregnant and postpartum patients, it is possible that disparities might also exist in postpartum care for patients who experience OASIs. There are very limited data assessing access to specialized, focused postpartum care for patients who have experienced an OASI or who have other postpartum pelvic floor concerns (20).

The purpose of this study was to evaluate disparities in and potential barriers to accessing care in a PPFHC utilizing a historical cohort for comparison of sociodemographic factors. We hypothesized that patients from more resource-deprived neighborhoods with higher area deprivation indices (ADI) would be less represented within a specialty PPFHC patient population compared to a historical cohort of OASIs.

## 2 Materials and methods

We performed a retrospective comparative cohort study of two pre-existing datasets, which were both chosen as convenience samples from a large academic hospital. The first dataset was a pre-PPFHC historical cohort of all OASIs from December 2019 to January 2021 before the institutional creation of the specialized PPFHC clinic. These patients experienced an OASI at that time of vaginal delivery and received routine postpartum care from a physician, nurse midwife, or advanced practice provider within their primary obstetric provider group. Patients in this cohort had been identified by documentation of either a third- or fourth-degree perineal laceration in the delivery records. Demographic, delivery, and postpartum data were subsequently abstracted from the electronic medical record.

The second dataset consisted of all patients who were evaluated in a specialized PPFHC for evaluation after a vaginal delivery from July 2021 to July 2022. The PPFHC is a specialized clinic staffed by board-certified urogynecologists. Patients are referred to the PPFHC at the discretion of their primary obstetric provider for postpartum care of OASIs, other complex obstetrical lacerations, or postpartum pelvic floor disorders including but not limited to wound healing issues, urinary and anal incontinence, and pain. Patients in this cohort were identified if they had been evaluated in the PPFHC. Demographic, delivery, and postpartum data were subsequently abstracted from the electronic medical record. For both cohorts, patients were excluded if their electronic health records were missing address data or if they resided in a state outside our normal referral base which includes Pennsylvania, Ohio, and West Virginia.

Our primary outcome involved the area deprivation index (ADI) (Neighborhood Atlas, University of Wisconsin), in which higher decile neighborhoods are considered more resource-deprived (21, 22). Patient addresses and zip codes were entered into the ADI database for Pennsylvania, West Virginia, and Ohio, and both state decile and national percentile rankings were recorded. If we assume equitable access to care, then sociodemographic factors, including the ADI of patients in the PPFHC, should be similar to those in the historical cohort. However, we hypothesized that there would be disparities in access to care in the PPFHC. Moreover, we hypothesized that the neighborhood ADI would be lower (less resource-deprived) among the PPFHC and patients from more resource-deprived neighborhoods would be underrepresented in the PPFHC compared to the historical cohort.

The secondary outcomes included the median household income (2020 American Community Survey, U.S. Census Bureau), for which patient zip codes were cross-referenced to their corresponding zip code tabulation areas. This identifier was then queried in the American Community Survey to collect median household income for that location. We also assessed driving distance (miles) and driving time (min) to the hospital (ArcMap GIS, ESRI, Redlands, California). The straight-line distance was calculated by ArcMap GIS, where the destination location was the coordinates for the PPFHC and the origin location was each patient’s address. Driving distance and time were also calculated with this software using the same coordinates, with driving time based on the driving route used for the driving distance. We also assessed the patient’s age, race, ethnicity, marital status, and insurance type. Any identified disparity is also a potential barrier to care, and thus by assessing disparities in the above sociodemographic primary and secondary outcomes, we are also assessing potential barriers to care.

Continuous variables were analyzed with independent *t*-tests or Mann–Whitney *U* tests, and categorical variables were analyzed by chi-square or Fisher’s exact tests using the SPSS statistical software (Version 28; IBM, Armonk, NY). To control for baseline differences between groups, we performed multivariable linear regression for continuous variables and logistic regression analyses for binary variables. Secondary to data skewness, the driving distance variable was log-transformed for the linear regression model. As this was a convenience sample from two previously collected datasets, no *a-priori* sample size calculation was performed. The University of Pittsburgh Institutional Review Board approved this research with a waiver of informed consent.

## 3 Results

There were 191 patients in the pre-PPFHC cohort and 263 patients in the PPFHC cohort (**Table 1**). Patients in the PPFHC cohort were older (31.3 vs. 29.9 years, *p* < 0.01) and more likely to be multiparous (20.3% vs. 13.1%, *p* = 0.04) than the historical cohort. There were no differences in race, ethnicity, insurance type, marital status, or gestational age at delivery between cohorts (**Table 1**). The historical cohort had a greater proportion of OASI owing to the data collected within the existing database and the broader referral patterns for the PPFHC; however, the rates of operative vaginal deliveries were similar between groups. Patients in the PPFHC cohort had significantly more postpartum visits than pre-PPFHC patients [median 3 (IQR 2–4) vs. 2 (IQR 1–2) visits, *p* = 0.01] (**Table 2**).

**Table 1:** Demographic and delivery characteristics.

Variable Name	Historical cohort of obstetrical anal sphincter injuries (*n* = 191)	Postpartum pelvic floor healing clinic cohort (*n* = 266)	*p*-value
Age (years)	29.9 ± 4.9	31.3 ± 4.2	<0.001
Race			0.53
White	145 (75.9%)	186 (69.9%)	
Black	9 (4.7%)	11 (4.1%)	
Asian	30 (15.7%)	52 (19.5%)	
American Indian, Alaska Native, Native Hawaiian, or Pacific Islander	1 (0.5%)	2 (0.8%)	
Unknown or not reported	6 (3.1%)	15 (5.6%)	
Ethnicity			0.83
Not Hispanic or Latino	178 (93.2%)	244 (91.7%)	
Hispanic or Latino	3 (1.6%)	4 (1.4%)	
Unknown or not reported	10 (5.2%)	18 (6.8%)	
Gestational weeks at delivery	39 (39–40)	39 (39–40)	0.68
Birthweight (g)	3,517 ± 434	3,409 ± 450	0.01
Operative vaginal delivery	65 (34.4%)	89 (34.1%)	0.95
Operative delivery type			0.14
Vacuum	34 (52.3%)	36 (40.4%)	
Forceps	31 (47.7%)	53 (59.6%)	
Parity >1	25 (13.1%)	54 (20.3%)	0.04
Marital status			0.15
Married	140 (76.5%)	206 (82.1%)	
Single	43 (23.5%)	45 (17.9%)	
Insurance type			0.72
Private	161 (85.2%)	228 (85.7%)	
Medicare or Medicaid	24 (12.7%)	35 (13.2%)	
Other	4 (2.1%)	3 (1.1%)	
Vaginal laceration type			<0.001
None	0 (0.0%)	10 (3.8%)	
1st degree	0 (0.0%)	5 (1.9%)	
2nd degree	0 (0.0%)	46 (17.3%)	
3rd degree	174 (91.1%)	179 (67.3%)	
4th degree	17 (8.9%)	26 (9.8%)	

Data presented as *N* (%), mean ± standard deviation, or median (interquartile range).

**Table 2:** Primary and secondary outcome data.

Variable name	Historical cohort of obstetrical anal sphincter injuries (*n* = 191)	Postpartum pelvic floor healing clinic cohort (*n* = 266)	*p*-value
ADI decile	5.0 (3.0–7.0)	4.0 (2.0–7.0)	0.06
Categorical ADI decile			0.06
1 (least deprived)	18 (9.5%)	39 (14.8%)	
2	28 (14.7%)	40 (15.2%)	
3	28 (14.7%)	34 (12.9%)	
4	17 (8.9%)	31 (11.7%)	
5	25 (13.2%)	35 (13.3%)	
6	12 (6.3%)	16 (6.1%)	
7	18 (9.5%)	27 (10.2%)	
8	18 (9.5%)	15 (5.7%)	
9	9 (4.7%)	15 (5.7%)	
10 (most deprived)	17 (8.9%)	12 (4.5%)	
ADI percentile	55 (36–73)	53 (32.5–69)	0.08
Median income (dollars)	63,598 (54,842–77,026)	70,756 (52,450–85,097)	0.38
Categorical median income			0.21
<$50,000	29 (15.2%)	50 (18.8%)	
$50,000–75,000	107 (56%)	122 (45.9%)	
$75,000–100,000	35 (18.3%)	60 (22.6%)	
>$100,000	20 (10.5%)	34 (12.8%)	
Straight-line distance (miles)	9.6 (5.5–17.0)	8.1 (4.9–13.5)	0.02
Driving distance (miles)	12.3 (7.0–21.1)	10.8 (6.3–17.2)	0.02
Driving time (min)	28.0 (18.7–44.2)	25.2 (16.7–35.5)	0.03
Number of postpartum visits	2 (1–2)	3 (2–4)	<0.001

Data presented as *N* (%) or median (interquartile range). Distance refers to the distance from the patient’s home to the hospital.

ADI, area deprivation index.

In the analysis of patient ADIs, no statistically significant difference was found between cohorts (**Table 2**). There were non-significant trends toward lower (less deprived) ADI values in the PPFHC; similarly, fewer patients from more deprived neighborhoods were seen in the PPFHC compared to the historical cohort (**Table 2**). Half as many patients from the most deprived neighborhoods (highest ADI decile) were seen in the PPFHC cohort compared to the pre-PPFHC cohort (4.5% vs. 8.9%, **Table 2**). On multivariable linear regression for ADI controlling for age, marital status, and obstetrical laceration type, our findings were unchanged: ADI was not significantly different between the PPFHC and historical pre-PPFHC cohorts (*B* = −0.19, 95% CI −0.70 to 0.31, *p* = 0.46). Lastly, we dichotomized the ADI deciles into least deprived (deciles 1–6) and most deprived (deciles 7–10) and re-ran our statistical analyses with a multivariable logistic regression, which did not change our results with no significant difference in dichotomized ADI between cohorts (OR 0.82, 95% CI 0.51 to 1.32, *p* = 0.42).

Patients in the PPFHC cohort lived closer to the hospital by a straight-line distance of 1.52 miles [pre-PPFHC: 8.08 (IQR 4.90–13.45) vs. 9.60 (IQR 5.48–16.96) miles, *p* = 0.02]. Driving distance was also significantly shorter in the PPFHC cohort [10.8 (IQR 6.3–17.2) vs. 12.3 (IQR 6.9–21.1) miles, *p* = 0.02]. Similarly, the associated driving time was significantly shorter for the PPFHC cohort [25.2 (IQR 16.7–35.5) vs. 27.96 (IQR 18.7–44.2) min, *p* = 0.03]. After performing a multivariable linear regression controlling for patient age, driving distance was no longer significantly different between groups (*B* = 0.13, 95% CI −0.03 to 0.29, *p* = 0.10).

## 4 Discussion

There were no significant differences in neighborhood ADI, median income, driving distance, race, ethnicity, or insurance type among patients seen in a specialized PPFHC compared to a historical cohort of OASIs. Our data are reassuring and suggest that patients who are at increased risk for postpartum pelvic floor disorders are able to access care in a PPFHC, regardless of these sociodemographic and socioeconomic factors. While not statistically significant, trends in our data suggest that access to care in the PPFHC may be more difficult for patients from more resource-deprived neighborhoods, and we should continue to monitor access for these populations.

The recommended postoperative care of OASIs includes early and close follow-up (23). Owing to both the short-term and long-term risks associated with OASIs, many institutions have moved to a model that offers a dedicated clinic setting for the follow-up of complex obstetrical lacerations (7). These dedicated pelvic floor clinics have been associated with high patient satisfaction (24) and provide an opportunity for early identification and treatment of postpartum pelvic floor disorders. However, there are limited data assessing patient access to this specialized type of care (25). Our results are reassuring in that there were no sociodemographic differences among patients who accessed the PPFHC compared to a historical cohort of women with higher-order obstetrical lacerations. Importantly, there were no differences in racial or ethnic proportions between the two cohorts. This is an important finding given the disproportionate disparities that Black women in the US face throughout obstetrical care, labor, and delivery (26).

As specialized postpartum pelvic floor healing clinics continue to emerge, it is important to pay attention to equitable access to care. While this seems intuitive, we do not want to recreate inequities that have long persisted in maternal healthcare (25–27). While our results are reassuring, non-significant trends in our data suggest that we need to monitor access for patients from more resource-deprived neighborhoods to ensure they continue to receive the postpartum pelvic floor care that is necessary. Other specialized pelvic floor healing clinics in the United States may use these data as a model to track access to care and assess whether care in similar clinics around the country is equitable. Lastly, while not significant on regression modeling, patients who lived farther from the hospital were underrepresented in the PPFHC cohort on unadjusted analyses. Potential solutions to overcome distance as a potential barrier to care may include telehealth visits with the ability to securely submit photos through the medical record if wound concerns exist. Another potential solution is offering services at satellite office locations closer to patient communities. While not studied specifically as part of this research, we have found that patients will often schedule PPFHC appointments in outreach locations closer to their homes (farther from the hospital of delivery) when this option is available.

We demonstrate that patients who attended the PPFHC had significantly more postpartum visits than the historical cohort of OASIs. Prior data demonstrate that, overall, only half of mothers attend a postpartum visit (27). While this may not be clinically impactful for patients with lesser-degree obstetrical lacerations, patients with OASIs should receive closer follow-up (7). A dedicated PPFHC may be a way to encourage adequate follow-up for a patient population at high risk for a variety of postpartum pelvic floor disorders.

The current study represents data from one institution, and it is important that providers who offer specialized postpartum pelvic floor care also assess their own patient populations to identify potential inequities and barriers to access to care. An area to monitor is our identified trend toward patients from more resource-deprived areas being less represented among the PPFHC cohort. Though not significant, this suggests that there is potential to miss a critical population should additional barriers arise and are not adequately identified and addressed. Future research in postpartum care clinics should assess equitable access or potential disparities in different geographic areas and in larger patient populations.

The strengths of our study include data from a large institution that evaluates and treats patients from both urban and rural settings, making our results generalizable to the greater population. We also have a historical cohort of OASIs available, which represents a patient population at increased risk of postpartum pelvic floor concerns and can thus be used for comparison to the PPFHC, which represents a patient population with a similarly increased risk of postpartum pelvic floor disorders (7). By using a historical cohort, we remove the possibility of bias related to patient choice or differing referral patterns between groups, as the historical cohort did not have the option to attend or be referred to a PPFHC. Additionally, we utilized software that provides tangible estimates for socioeconomic status to the best of our abilities given our retrospective data.

Our study has important limitations. The two patient cohorts differ in that the historical cohort consisted solely of OASIs, whereas the PPFHC cohort also had a small proportion of non-OASI patients. However, the majority of patients (77%) in the PPFHC had OASIs; thus, both groups remain comparable and represent patients at an increased risk of postpartum pelvic floor complications and disorders. We also recognize that while our study evaluates area deprivation index, time, distance, and median household income, there are several other sociodemographic variables that can contribute to a patient’s ability to access care including but not limited to employment, education level, and housing. Additionally, we demonstrate a non-significant trend, suggesting that access to a postpartum pelvic floor healing clinic may be more difficult for patients from more resource-deprived neighborhoods. Thus, it is possible that with a larger sample size, a significant difference might emerge, supporting similar studies among larger patient populations. The generalizability of our study is also limited given the use of a historical cohort and the data were derived from a single center. Our inclusion and assessment of driving time was predicated on the assumption that every patient has access to a car. However, we know that this is not the case, and we were not able to assess accessibility to transportation or public transportation routes, which would impact access. Additionally, the ArcGIS software used for calculating distance and time of travel to the clinic assumes a singular route, when in reality, there are several possible routes. Lastly, we have a small number of Black patients in our cohort, and thus, it is difficult to draw conclusions regarding racial inequities from our data. Prior data demonstrate that OASIs occur less frequently in Black patients, and thus, our small number of Black patients is consistent with prior research (28).

## Data Availability

The raw data supporting the conclusions of this article can be made available by the authors after discussion and with a data usage agreement.

## Author’s note

This research was presented as an abstract presentation during the American Urogynecologic Society (AUGS) Pelvic Floor Disorders Week in Portland, Oregon, October 4-6, 2023.
